# The Wheat Nitro-Proteome: Protein Nitration Profiles During Drought and Rehydration

**DOI:** 10.3390/plants15131951

**Published:** 2026-06-24

**Authors:** Marta Gietler, Justyna Fidler-Jarkowska, Małgorzata Nykiel

**Affiliations:** Department of Biochemistry and Microbiology, Institute of Biology, Warsaw University of Life Sciences-SGGW, Nowoursynowska 159, 02-776 Warsaw, Poland; justyna_fidler@sggw.edu.pl (J.F.-J.); malgorzata_nykiel@sggw.edu.pl (M.N.)

**Keywords:** drought, *Triticum aestivum* L., nitrosative stress, protein nitration, rehydration

## Abstract

Protein nitration within the nitro-proteome is a dynamic component of drought and recovery responses in wheat (*Triticum aestivum* L.), yet its role in stress adaptation remains unclear. Young wheat seedlings demonstrate a degree of drought resistance, characterized by physiological and morphological adaptations, during the initial growth phases. However, this tolerance begins to diminish significantly in 5-day-old seedlings. The mechanisms behind this phenomenon are unclear. Our results indicate that it may be related to protein nitration. This study compared the physiological and nitrosative responses of 4-day-old drought-tolerant and 6-day-old sensitive wheat seedlings subjected to drought followed by rehydration. In tolerant seedlings, in contrast to sensitive ones, the water saturation deficit after rehydration returned to the control levels, confirming their drought tolerance. Moreover, NO_2_^−^ accumulation in the recovery group was significantly higher in sensitive seedlings than in the control group. Results indicate that drought resistance correlates with protein nitration during the recovery phase. Nitro-proteomic analysis revealed that in tolerant seedlings, protein nitration is limited. The most significant differences are observed in the recovery group, with predominant downregulation of protein nitration in tolerant seedlings and significant upregulation of numerous proteins in sensitive seedlings. Upregulated nitration of vital proteins involved in energy production, photosynthesis (such as the Rubisco large subunit), ATP synthases, and cytosolic malate dehydrogenase may lead to disturbances in energy metabolism and thus prevent an effective response to changing environmental conditions. These findings suggest that regulation of protein nitration during recovery may contribute to drought resilience in wheat and could represent a potential target for improving stress tolerance.

## 1. Introduction

Nitric oxide (NO) is a ubiquitous signaling molecule that regulates plant responses to environmental stresses and developmental processes, including defense against pathogens, tolerance to drought, salinity, and temperature extremes, as well as seed dormancy, germination, and senescence [1]. Directly or indirectly, it modifies protein structure, leading to the nitration of tyrosine, tryptophan, and histidine residues, or the S-nitrosylation of thiol groups [2,3]. While the S-nitrosylation of spring wheat proteins under water-deficiency conditions has been well characterized [4], the nitration profile of plant proteins remains poorly understood. This gap highlights the novelty and significance of the present study within this experimental model.

Protein nitration is an important post-translational modification that regulates protein structure and function in plant cells. The recent literature primarily describes the nitration of tyrosine residues, a covalent modification in which a nitro group is added to an aromatic ring. The nitration mechanism is a two-stage process. In the first stage, nitric oxide (NO•) and superoxide anion (O_2_•^−^) react to form peroxynitrite (ONOO^−^), which reacts with the tyrosine residue to form an unstable tyrosyl radical. In the second stage, the tyrosyl radical is converted to 3-nitrotyrosine (3-NT) under the influence of reactive nitrogen species. Tyrosine nitration is a potential molecular switch that can modulate enzyme activity, alter protein conformation, and interfere with downstream signaling cascades. However, it is also a marker of nitrosative stress. Distinguishing whether nitration of a given protein is a biomarker or a precise regulatory mechanism presents a significant challenge in plant biology. An increase in 3-NT accumulation serves as an excellent chemical indicator of an altered cellular redox state, reflecting elevated levels of ONOO^−^ [5]. Demonstrating a regulatory mechanism associated with nitration requires biochemical validation to prove that modification of a specific tyrosine residue alters enzyme kinetics or substrate affinity. In plants, only a limited number of proteins have been definitively proven to undergo direct functional transitions upon nitration [6,7,8].

Nitration is considered a highly selective modification, dependent on factors such as the protein’s structure and location [9]. Certain proteins, especially those found in chloroplasts and mitochondria, are particularly vulnerable to nitration. This susceptibility can significantly impact the fundamental energy metabolism of plants. Recent research shows that protein nitration significantly influences key physiological processes and plant ontogenetic development [10]. In animals, protein nitration is a reversible process that can compete with phosphorylation in the signal transduction pathway; however, the possibility of denitration in plants remains unknown [11,12]. It is currently also considered a marker of nitrosative stress in plants [13]. Furthermore, a significant contribution of nitrated proteins to stress-induced signal transduction, including in response to water deficiency, has been demonstrated. The available literature contains reports of increased nitration due to a water deficit, for example, in the roots of *Lotus japonicus* [14]. It has also been demonstrated that pretreatment of citrus fruits with nitric oxide increases their drought tolerance, suggesting that this modification may contribute to acclimation mechanisms [15].

Nitration generally has a detrimental effect on the function of target proteins. However, there are noteworthy instances in which it either has no impact or even enhances protein functionality [16]. It is important to note that tyrosine nitration is a highly specific process influenced by various protein characteristics, such as quaternary structure and environmental factors, as well as by whether the protein is soluble or membrane-bound [5,17]. Changes in nitrated protein abundance may reflect stress-induced remodeling of metabolic pathways and adjustments aimed at maintaining cellular homeostasis under adverse conditions.

Wheat (*Triticum aestivum* L.) seedlings are capable of enduring a significant water deficit until about the fifth day of germination. However, the emergence of the first leaf from the coleoptile marks a shift towards increased vulnerability to dehydration [18]. This transition from tolerance to sensitivity in seedlings is believed to be primarily linked to alterations in the hexose/sucrose balance, which reflects the “cellular energy state” in the developing seedlings of monocots and legumes [19,20]. Moreover, our previous research showed that redox homeostasis [21] and post-translational protein modifications, such as carbonylation [22], S-nitrosation, and S-glutathionylation [4], are also linked to the shift in drought susceptibility.

According to our hypothesis, drought tolerance in wheat seedlings is linked to the developmental stage-dependent regulation of reactive nitrogen species metabolism and protein nitration. Specifically, we propose that 4-day-old drought-tolerant seedlings and 6-day-old drought-sensitive seedlings of the same wheat cultivar differ in nitro-proteome composition, water saturation deficit (WSD), and nitrate/nitrite accumulation under drought conditions and during recovery. These differences are expected to reflect developmental plasticity of a single genome rather than genotypic variation, thereby enabling the identification of key molecular mechanisms associated with drought adaptation.

The use of seedlings of different ages from the same cultivar provides a unique experimental model that eliminates genetic background effects and allows for an analysis of stress responses driven solely by developmental regulation. Comparative analysis of nitrated proteins using 2DE-based nitro-proteomics, together with physiological and biochemical parameters, may help identify protein nitration-related processes associated with drought tolerance and recovery capacity in wheat.

## 2. Results

### 2.1. Water Saturation Deficit in Tolerant and Sensitive Plants Under Drought and Recovery Conditions

**Figure 1:**
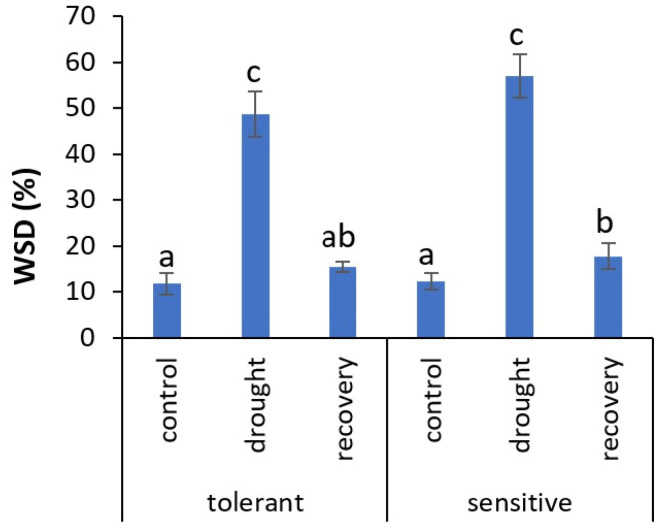
Water saturation deficit (%). Bars represent mean ± SD. Letters indicate homogeneous groups according to Tukey’s test (α = 0.05).

WSD varied between 4-day-old drought-tolerant seedlings and 6-day-old drought-sensitive seedlings. Under control conditions, both groups exhibited similar WSD levels, approximately 12%. This initial uniformity establishes that, prior to stress exposure, both developmental groups had identical physiological water status, confirming that subsequent variations were strictly stress-induced rather than developmentally predetermined. However, drought significantly increased WSD in both groups, reaching approximately 48% in drought-tolerant seedlings and 58% in drought-sensitive seedlings. After rewatering, WSD decreased in both groups to below 20%; however, in 6-day-old seedlings, it remained statistically higher than the control group, whereas in tolerant seedlings, WSD in the recovery group was not significantly different from the control. The incomplete recovery of hydration in 6-day-old seedlings during the rewatering phase suggests impaired restoration of cellular homeostasis, potentially associated with deregulated reactive nitrogen species metabolism and protein nitration (Figure 1).

### 2.2. Changes in Nitrate (NO_3_^−^) and Nitrite (NO_2_^−^) Content Under Drought Stress Recovery Conditions

The comparison of nitrate and nitrite ion content in plants under control, drought, and recovery conditions indicated that water deficit leads to the accumulation of nitrogen-containing compounds, particularly NO_2_^−^ ions, which are associated with toxic and nitrosative effects.

**Figure 2:**
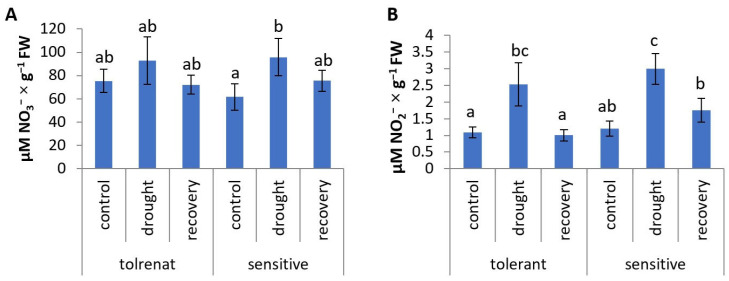
Nitrate (**A**) and nitrite (**B**) ion contents in tolerant and sensitive seedlings. Bars represent mean ± SD. Letters indicate homogeneous groups according to Tukey’s test (α = 0.05).

Under drought conditions, NO_3_^−^ levels increased in both genotypes. In drought-tolerant seedlings, nitrate content increased by approximately 22.7% under drought conditions, while in drought-sensitive seedlings, the increase was more pronounced, reaching about 53.2%. The initial NO_3_^−^ content under control conditions was slightly lower in 6-day-old sensitive seedlings compared to 4-day-old tolerant seedlings; however, this difference was not statistically significant (Figure 2A).

A similar trend was observed for NO_2_^−^ ions. In 4-day-old drought-tolerant seedlings, the NO_2_^−^ content increased by approximately 127%, whereas in 6-day-old drought-sensitive seedlings, the increase reached about 150%. Despite the higher accumulation observed in sensitive plants, differences in NO_2_^−^ content between tolerant and sensitive groups were not statistically significant (Figure 2B).

Following the rehydration phase, clear differences in recovery capacity between the drought-tolerant and drought-sensitive seedlings were observed. In drought-tolerant seedlings, NO_2_^−^ levels returned to levels comparable to those in the control, indicating effective restoration of nitrogen homeostasis [23]. In contrast, drought-sensitive seedlings in the recovery group had significantly higher NO_2_^−^ content than tolerant seedlings. This elevated nitrite content may suggest prolonged nitrosative stress in sensitive plants, which can be formed through the conversion of reactive nitrogen species such as nitrogen dioxide NO_2_ and dinitrogen trioxide N_2_O_3_, which are generated under conditions of elevated NO accumulation [24] (Figure 2B).

### 2.3. Principal Component Analysis (PCA) of Tyrosine-Nitrated Proteomes Under Drought and Rehydration

The PCA revealed distinct clustering patterns between developmental stages and treatment conditions, suggesting differences in nitro-proteome plasticity.

PCA showed that the proteomic profiles of tyrosine-nitrated proteins in 4-day-old seedlings from the control, drought, and rehydration groups differed in both PC1 (34.020% variability) and PC2 (19.825% variability), constituting separate sets. Clear separation between control, drought, and rehydration samples suggests a more dynamic and reversible adjustment of protein nitration profiles in response to changing environmental conditions.

**Figure 3:**
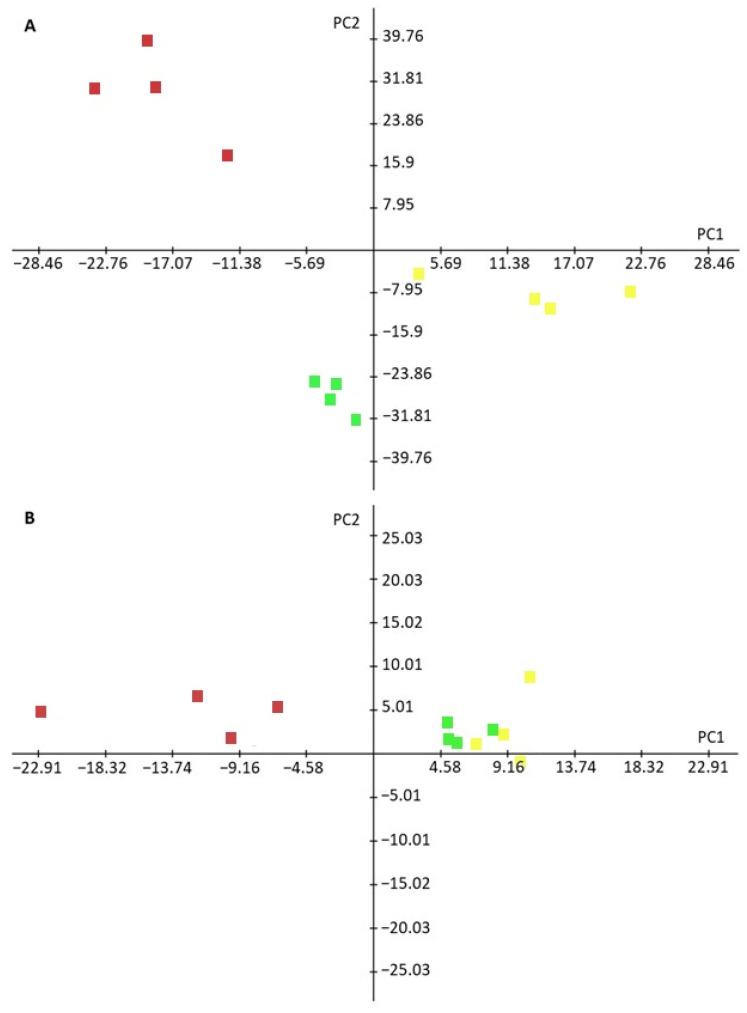
Principal component analysis of Western blot protein spot intensities. (**A**) Drought-tolerant wheat seedlings; (**B**) drought-sensitive wheat seedlings. The analysis was performed based on normalized spot intensity values obtained from Delta2D image analysis of Western blot membranes. Spot intensities were normalized to the total intensity of all detected spots in each membrane. Red indicates control conditions, green indicates drought treatment, and yellow indicates recovery after rehydration. Each point represents an independent biological replicate.

In 6-day-old seedlings, the same analysis revealed no significant differences in PC1 (34.973% variability) between drought- and rehydration-treated seedlings, whereas the control group formed a separate set. No significant differences in PC2 (22.014% variability) values were observed between the groups (Figure 3). This may indicate a reduced capacity to fully re-establish the control-like proteomic state after stress. This pattern could be consistent with a more constrained or less reversible response of the nitro-proteome to drought and recovery.

### 2.4. Proteomic Insights into Tyrosine Nitration Under Drought and Recovery

#### 2.4.1. Photosynthesis and Calvin Cycle: Major Targets of Nitration

In tolerant seedlings, under drought stress, we observed a decrease in the abundance of the nitrated Rubisco large subunit, which may indicate a modulation of carbon fixation capacity under water deficit conditions, whereas Rubisco activase A increased, which could reflect a stress-related adjustment of Rubisco activation dynamics. Other nitrated Calvin cycle enzymes, including sedoheptulose-1,7-bisphosphatase and phosphoglycerate kinase, were reduced, suggesting a downregulation or remodeling of photosynthetic carbon metabolism under drought stress. During recovery, an increase in nitrated Rubisco activase and OEE2, which are photosystem II-associated components, may indicate the re-establishment and fine-tuning of photosynthetic electron transport and Calvin cycle regulation upon rehydration. Overall, these changes appear to be associated with the transition between drought and recovery treatments and may reflect dynamic regulation of photosynthetic and energy-related processes in response to changing water availability (Table 1).

In sensitive seedlings, drought caused a stronger reduction in nitration of key photosynthetic proteins, including phosphoribulokinase and ferredoxin–NADP reductase. During recovery, several Calvin cycle enzymes showed increased nitration, including Rubisco activase, sedoheptulose-1,7-bisphosphatase, and aldolase 8 (Table 2).

#### 2.4.2. Energy Metabolism: Nitration of ATP Synthase Complexes

ATP synthase complexes were highly responsive to drought-induced nitration, with distinct responses between tolerant and sensitive seedlings.

In tolerant seedlings, nitrated chloroplast ATP synthase subunits decreased, but the nitrated mitochondrial ATP synthase α subunit increased (Table 1). In sensitive seedlings, nitrated mitochondrial ATP synthase subunits showed variable responses during drought, indicating limited metabolic flexibility. During recovery, strong upregulation of nitrated chloroplast ATP synthase subunits was observed (Table 2).

#### 2.4.3. Stress Response and Antioxidant System

In tolerant seedlings, nitrated MDAR6 and 2-Cys peroxiredoxin BAS1 were downregulated during drought but showed partial recovery during rehydration to control levels. Nitrated HSP70 proteins remained moderately reduced (Table 1).

In sensitive seedlings, stronger suppression of nitration of detoxification-related proteins was observed during drought. Aldehyde dehydrogenase 7B1 showed a decrease in nitration under stress but a pronounced increase during recovery (Table 2).

#### 2.4.4. Signaling and Translation

In tolerant seedlings, the 14-3-3 protein showed a slight decrease in nitration during drought, followed by strong upregulation during recovery. Similarly, nitrated SAM synthase increased under drought and further increased during recovery. Nitrated translation initiation factor 5A2 was also strongly upregulated during drought (Table 1).

In sensitive seedlings, downregulation of nitration of metabolic regulators was observed during drought, including cytosolic malate dehydrogenase and plastid glutamine synthetase 2. During recovery, an increased abundance of nitrated actin was observed (Table 2).

**Table 1:** Differentially abundant nitrated proteins in sensitive seedlings identified from selected spots by LC–MS/MS in 4-day-old *Triticum aestivum* (L.) seedlings. Abbreviations: ID—number assigned to spot; Ratio d/c—abundance of proteins in drought conditions compared to control; Ratio r/c—abundance of proteins in recovery compared to control; Seq—sequences; emPAI—exponentially modified protein abundance index; pI—isoelectric point; Cov—coverage (%).

Tolerant Seedlings											
ID	Ratio d/c	Ratio r/c	Protein	Accession	Score	Matches	Seq	emPAI	pI	Mass	Cov
748	0.831	0.531	ATP synthase CF1 beta subunit (chloroplast)	NP_114266.1	2262	26(26)	11(11)	1.82	5.06	53,881	29
762	0.803	0.606	ribulose-1,5-bisphosphate carboxylase/oxygenase large subunit (chloroplast)	NP_114267.1	712	16(16)	10(10)	1.23	6.22	53,445	20
869	0.284	0.317	chaperone protein ClpC1, chloroplastic	XP_020167125.1	6114	104(104)	43(43)	5.44	5.99	101,630	43
929	0.581	0.446	ATP synthase alpha subunit (chloroplast)	AAA84725.1	673	12(12)	9(9)	0.99	5.94	55,287	19
933	0.433	0.288	ATP synthase CF1 alpha subunit (chloroplast)	NP_114256.1	795	14(14)	10(10)	1.17	6.11	55,318	19
965	0.315	0.568	delta 1-pyrroline-5-carboxylate synthetase	AAX35536.1	1457	26(26)	16(16)	1.38	6.14	78,222	26
1019	0.719	0.492	subtilisin protease	ACB87529.1	999	16(16)	10(10)	1.05	5.12	59,593	18
1054	0.46	0.524	HSP70	AAB99745.1	4570	74(74)	24(24)	3.27	5.14	71,385	38
1059	0.622	0.547	HSP70	AAB99745.1	5587	94(94)	26(26)	4.12	5.15	71,386	43
1176	0.401	0.72	beta-glucosidase 1b, Precursor	Q1XH05.1	2623	45(45)	20(20)	2.67	5.67	64,898	33
1183	0.282	0.46	aldehyde dehydrogenase 7B1	AKE36953.1	2773	45(45)	12(12)	2.23	6.12	55,092	24
1276	2.03	1.418	ATP synthase subunit alpha, mitochondrial	P12862.1	1030	18(18)	15(15)	2.15	5.70	55,515	28
1304	0.676	0.541	adenosylhomocysteinase	SPT20281.1	1206	20(20)	14(14)	2.03	5.62	53,905	29
1349	0.551	0.741	ATP synthase beta subunit	CAA52636.1	14,592	246(246)	22(22)	4.25	5.56	59,326	47
1425	0.421	1.125	eukaryotic initiation factor eIF4A	AQU14667.1	5050	95(95)	24(24)	8.67	5.31	47,227	50
1431	0.383	0.903	chloroplast MDAR6 protein	AKA43771.1	1056	15(15)	10(10)	1.27	5.93	52,410	23
1480	1.197	2.836	S-adenosylmethionine synthase	B0LXM0.1	1153	17(17)	8(8)	1.20	5.55	43,609	31
1537	0.626	1.225	phosphoglycerate kinase, chloroplastic; precursor	P12782.1	6016	99(99)	19(19)	3.56	6.58	49,980	38
1604	1.441	2.097	ribulose bisphosphate carboxylase/oxygenase activase A, partial	AIX47836.1	4542	61(61)	16(16)	4.43	5.35	42,255	45
1676	0.527	0.618	sedoheptulose-1,7-bisphosphatase, chloroplastic, precursor	P46285.1	4548	81(81)	16(16)	4.53	6.04	42,547	45
1820	0.286	0.538	fructose-1,6-bisphosphate aldolase 5	AVL25137.1	9879	161(161)	18(18)	5.70	6.78	42,072	50
1822	0.511	0.845	ferredoxin–NADP(H) oxidoreductase (plasmid)	CAD30025.1	1213	19(19)	14(14)	3.38	6.92	40,491	38
1864	0.399	0.481	ferredoxin–NADP(H) oxidoreductase (plasmid)	CAD30025.1	6375	114(114)	22(22)	13.15	6.93	40,492	53
2113	0.889	2.444	14-3-3 protein	AAR89812.1	882	14(14)	10(10)	3.28	4.83	29,388	35
2332	0.464	0.338	2-Cys peroxiredoxin BAS1, chloroplastic, precursor	P80602.2	3554	75(75)	12(12)	7.86	5.71	23,426	45
2348	0.513	0.687	2-Cys peroxiredoxin BAS1, chloroplastic, precursor	P80602.2	772	14(14)	6(6)	1.96	5.72	23,426	23
2420	0.576	1.808	oxygen-evolving enhancer protein 2, chloroplastic	Q00434.1	1945	40(40)	9(9)	3.06	8.84	27,424	26
2511	1.917	0.523	eukaryotic translation initiation factor 5A2	AAZ95172.1	987	16(16)	6(6)	3.22	5.70	17,576	35
9082	0.706	0.425	carbonic anhydrase	CDM83497.1	558	8(8)	6(6)	1.46	8.35	28,401	26

**Table 2:** Differentially abundant nitrated proteins in sensitive seedlings identified from selected spots by LC–MS/MS in 6-day-old *Triticum aestivum* (L.) seedlings. Abbreviations: ID—number assigned to spot; Ratio d/c—abundance of proteins in drought conditions compared to control; Ratio r/c—abundance of proteins in recovery compared to control; Seq—sequences; emPAI—exponentially modified protein abundance index; pI—isoelectric point; Cov—coverage (%).

Sensitive Seedlings											
ID	Ratio d/c	Ratio r/c	Protein	Accession	Score	Matches	Seq	emPAI	pI	Mass	Cov
948	0.907	1.477	ribulose-1,5-bisphosphate carboxylase/oxygenase large subunit (chloroplast)	NP_114267.1	1249	22(22)	9(9)	1.07	6.22	53,445	19
997	0.456	1.076	vacuolar proton-ATPase subunit A	ABD85016.1	754	9(9)	7(7)	0.55	5.23	68,754	13
1195	1.111	2.729	ATP synthase CF1 alpha subunit (chloroplast)	NP_114256.1	4141	75(75)	21(21)	4.10	6.11	55,318	41
1226	1.224	2.089	ATP synthase subunit alpha, mitochondrial	P12862.1	1514	26(26)	14(14)	1.96	5.70	55,515	27
1227	0.351	3.503	aldehyde dehydrogenase 7B1	AKE36953.1	1291	20(20)	10(10)	1.16	6.12	55,092	20
1235	0.997	1.573	ATP synthase subunit alpha, mitochondrial	P12862.1	4848	89(89)	21(21)	4.08	5.70	55,515	40
1237	0.526	1.281	ATP synthase subunit alpha, mitochondrial	P12862.1	4223	71(71)	22(22)	4.52	5.70	55,515	39
1259	0.545	1.322	ATP synthase CF1 beta subunit (chloroplast)	NP_114266.1	16,309	293(293)	21(21)	5.06	5.06	53,881	52
1401	1.121	2.396	RUBISCO activase alpha, partial	CDX48684.1	3476	39(39)	17(17)	3.99	5.62	44,839	47
1431	0.352	0.737	phosphoglycerate kinase, chloroplastic; precursor	P12782.1	1106	14(14)	10(10)	1.32	6.58	49,980	22
1441	0.604	1.680	actin	AHE76167.1	4594	100(100)	14(14)	3.62	5.31	41,849	41
1448	0.458	0.883	plastid glutamine synthetase 2	ACT22496.1	8602	159(159)	14(14)	2.87	5.75	47,002	34
1518	0.271	0.892	phosphoribulokinase, chloroplastic	CAB56544.1	12,037	198(198)	15(15)	3.50	5.72	45,512	36
1520	0.289	1.141	cytosolic malate dehydrogenase	ACQ57333.1	1132	14(14)	9(9)	1.92	5.75	35,831	35
1546	1.311	2.509	sedoheptulose-1,7-bisphosphatase, chloroplast precursor, expressed	CBH32512.1	2352	34(34)	10(10)	1.73	6.17	42,580	29
1558	0.246	0.579	sedoheptulose-1,7-bisphosphatase, chloroplastic; precursor	P46285.1	11,437	229(229)	17(17)	4.44	6.04	42,547	45
1564	0.616	0.746	fructose-1,6-bisphosphate aldolase 5	AVL25137.1	12,729	203(203)	14(14)	3.48	6.78	42,072	46
1570	0.679	2.038	fructose-1,6-bisphosphate aldolase 8	AVL25140.1	944	14(14)	8(8)	1.27	6.08	41,823	19
1571	0.456	0.855	fructose-1,6-bisphosphate aldolase 4	AVL25136.1	14,946	225(225)	14(14)	3.11	5.94	42,175	46
1625	0.318	0.359	ferredoxin–NADP(H) oxidoreductase (plasmid)	CAD30025.1	4583	90(90)	17(17)	6.49	6.92	40,491	43
1629	0.213	0.525	ferredoxin–NADP(H) oxidoreductase (plasmid)	CAD30025.1	9347	162(162)	18(18)	5.74	6.92	40,491	49
1926	0.703	1.749	oxygen-evolving enhancer protein 2, chloroplastic (precursor)	Q00434.1	14,610	254(254)	11(11)	4.46	8.84	27,424	37
2048	0.322	0.792	protein-L-isoaspartate O-methyltransferase	Q43209.1	1120	10(10)	4(4)	1.33	4.90	24,806	20

### 2.5. Proteomic Analysis of Drought Response and Recovery Based on Heatmap Profiling

**Figure 4:**
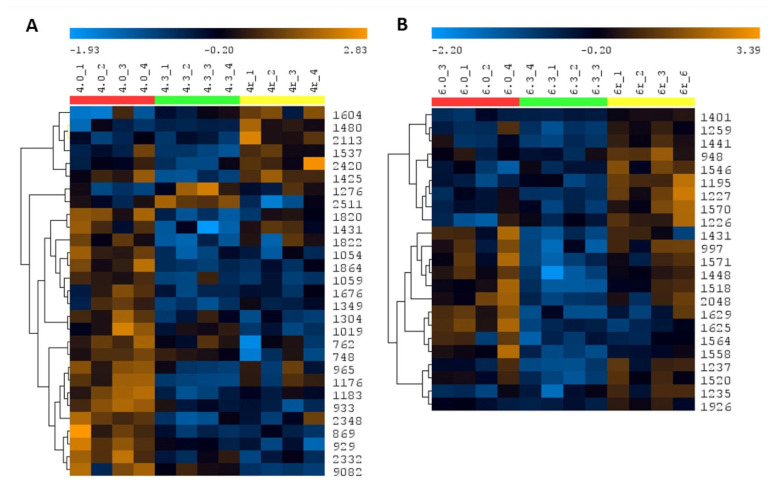
Heatmap and hierarchical clustering of differentially abundant proteins based on normalized spot intensities. (**A**) Drought-tolerant wheat seedlings; (**B**) drought-sensitive wheat seedlings. Red—control; green—drought treatment; yellow—recovery after rehydration. Each group consists of four biological replicates (*n* = 4). Proteins were selected based on one-way ANOVA with Bonferroni correction (*p* ≤ 0.05). Hierarchical clustering was performed using Euclidean distance to visualize protein abundance patterns across experimental treatments.

Heatmap analysis of nitrated protein abundance profiles in drought-tolerant (Figure 4A) and drought-sensitive seedlings (Figure 4B) revealed distinct patterns of protein accumulation in response to water deficit and subsequent recovery.

The hierarchical clustering heatmaps reveal distinct, contrasting tyrosine nitration profiles in tolerant (Figure 4A) and sensitive (Figure 4B) wheat seedlings across the three experimental conditions: control, drought, and recovery. During drought, the predominant change in tolerant seedlings was a systemic downregulation of protein nitration. Conversely, the sensitive 6-day-old seedlings exhibit a more diversified response under drought. The sensitive seedlings heatmap showed interspersed clusters of both upregulated and downregulated nitrated proteins. This contrast is further intensified during the recovery phase. The tolerant seedlings show increased nitration only in a subset of proteins after rehydration. In comparison, the sensitive seedlings heatmap shows several proteins with upregulated nitration during recovery. The heatmap depicts extensive protein clusters, indicating profoundly elevated nitration levels. This widespread post-drought nitration targets essential primary metabolic enzymes, such as ATP synthase and Rubisco.

### 2.6. Protein–Protein Interaction Network Analysis (STRING)

**Figure 5:**
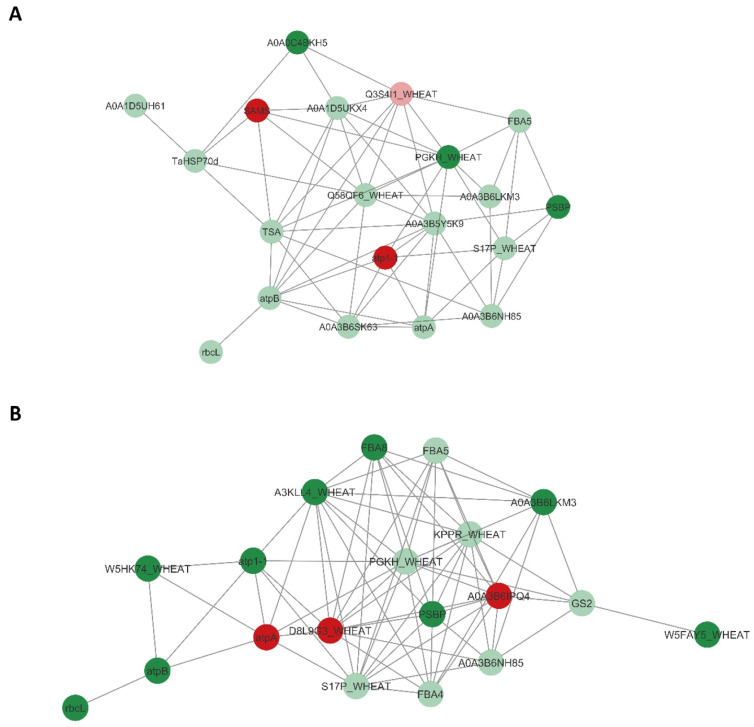
String network of nitrated proteins (Cytoscape 3.10). (**A**) Tolerant seedlings; (**B**) sensitive seedlings. Green—downregulated DAPs in drought; red—upregulated DAPs in drought; transparent—downregulated DAPs in recovery; opaque—upregulated DAPs in recovery. Protein–protein interaction networks were predicted using the STRING database (http://string-db.org, accessed on 2 April 2026), which integrates known and predicted interactions. Isolated nodes were removed from the final network visualizations.

Comparison of STRING interaction networks constructed for drought-tolerant (Figure 5A) and drought-sensitive seedlings (Figure 5B) revealed distinct differences in the organization of proteomic responses to drought stress and subsequent recovery. It should be noted that STRING networks are based on known and predicted protein–protein associations derived from multiple evidence sources and do not constitute direct experimental evidence of functional metabolic regulation. Therefore, the observed network organization should be interpreted as a putative representation of coordinated proteomic responses associated with the different treatments.

In both tolerant and sensitive seedlings, the central nodes of the interaction networks were associated with proteins involved in energy metabolism and photosynthesis. Based on the protein dataset, several major functional clusters were identified. A prominent interaction cluster corresponded to the ATP synthase complex, including chloroplastic CF1 α and β subunits (e.g., IDs 748, 929, 933, and 1349). These proteins displayed strong connectivity within the network. Another highly interconnected module corresponded to the Calvin–Benson cycle. Proteins such as the Rubisco large subunit (IDs 762 and 948), Rubisco activase (IDs 1604 and 1401), and fructose-1,6-bisphosphate aldolase (IDs 1820, 1564, 1570, and 1571) formed a dense interaction cluster, particularly evident in drought-sensitive seedlings.

Distinct differences in PPI STRING network organization were observed between drought-tolerant and drought-sensitive seedlings. In tolerant seedlings (Figure 5A), the network included proteins associated with protective and regulatory functions, such as HSP70 chaperones (IDs 1054 and 1059) and enzymes involved in metabolite biosynthesis, including S-adenosylmethionine synthase (ID 1480). A moderate density of interactions was observed in this network. In contrast, the network in sensitive seedlings (Figure 5B) showed increased connectivity among proteins involved in primary metabolism, particularly photosynthetic enzymes. Reduced abundance of key Calvin cycle proteins, such as sedoheptulose-1,7-bisphosphatase (ID 1558) and several aldolase isoforms, was observed under drought. The strong interconnections among these proteins suggest alterations in carbon assimilation pathways.

A substantial number of nitrated proteins that decreased in abundance during drought showed increased abundance during recovery. This trend was particularly evident in sensitive seedlings, where selected proteins displayed strong increases in nitration during recovery. In tolerant seedlings, most nitrated proteins in the network were downregulated in drought as well as in recovery.

### 2.7. Gene Ontology (GO) Analysis of Differentially Abundant Nitrated Proteins

**Figure 6:**
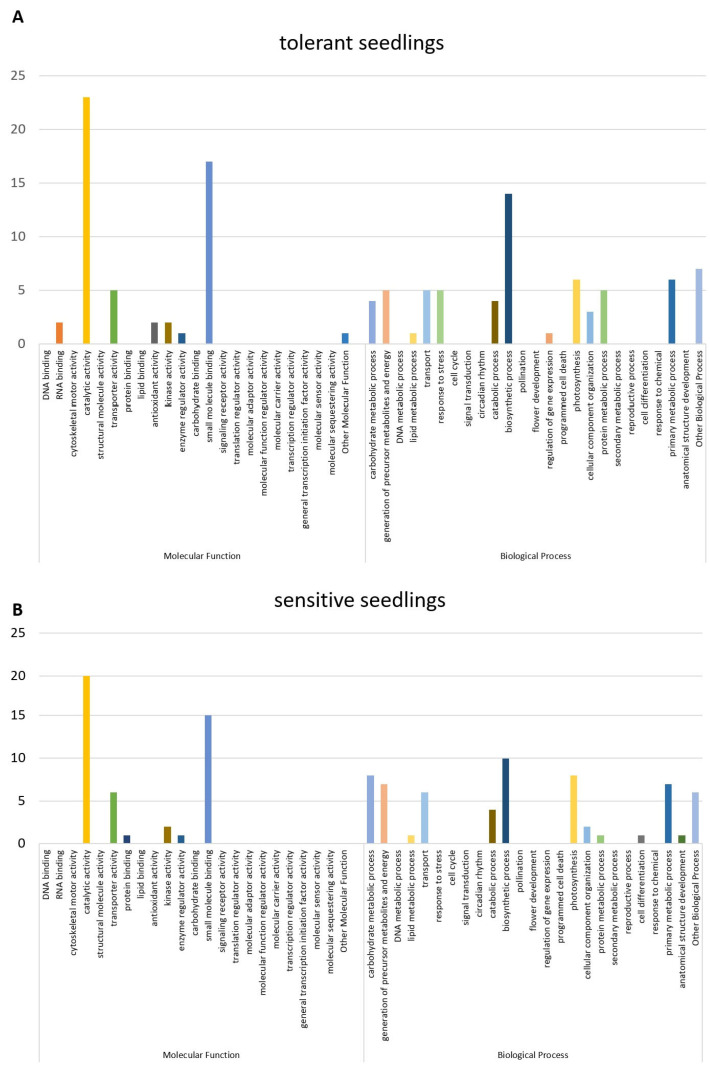
GO annotations of MF and BP categories for identified nitrated DAPs. (**A**) Drought-tolerant wheat seedlings; (**B**) drought-sensitive wheat seedlings. Functional annotation and GO term assignment were performed based on UniProt database information (UniProtKB). The analysis summarizes the distribution of identified proteins across GO categories for MF and BP.

The GO annotation analysis of nitrated proteins provides critical evidence of differences between tolerant and sensitive wheat seedlings. In the tolerant 4-day-old seedlings (Figure 6A), the nitro-proteome shows a high degree of functional diversity within the Biological Process (BP) category. There is a strong representation of proteins involved in biosynthetic processes, photosynthesis, protein metabolism, and primary metabolism. Furthermore, the unique presence of nitrated proteins associated with the response to stress was observed in 4-day-old seedlings. Under Molecular Function (MF), tolerant seedlings exhibited antioxidant activity, although the two main categories were catalytic activity and small-molecule binding. In contrast, the sensitive 6-day-old seedlings (Figure 6B) displayed a narrower BP functional scope, with the nitro-proteome primarily concentrated in biosynthetic process, carbohydrate metabolic process, and photosynthesis. The MF profile of the nitro-proteome also showed that nitrated proteins were concentrated in two main categories: catalytic activity and small-molecule binding. MF GO analysis of the sensitive plants reveals a notable absence of antioxidant activity and RNA binding in the nitro-proteome.

## 3. Discussion

The physiological response to drought is characterized by WSD values, which serve as direct indicators of a plant’s water status and cellular hydration levels. Both seedlings exhibited a sharp increase in WSD during the drought period, yet the sensitive 6-day-old seedlings reached a higher deficit than the tolerant 4-day-old seedlings, consistent with our previous work on a similar experimental model [4,21,22]. Together with the fact that nitrate ions significantly increased in response to drought in sensitive seedlings only, and that nitrite ions in the recovery group remained at a higher level in sensitive seedlings than in tolerant ones, it suggests that severe dehydration disturbs nitrogen metabolism [25]. The substantial water deficit in sensitive seedlings likely triggers a more intense nitrosative burst due to cellular desiccation and subsequent metabolic disruption.

The recovery phase highlights the dehydration tolerance of 4-day-old seedlings, which restored their water status to a level nearly identical to that of the control group. In contrast, the 6-day-old sensitive seedlings maintained a slightly higher residual WSD upon rehydration, paralleling the higher nitrite ion concentration in the sensitive seedlings of the recovery group. This persistent water deficit, even if minor, may contribute to sustained RNS levels and a higher degree of protein nitration, as discussed previously [26]. It seems that the tolerant seedlings’ ability to restore cellular hydration more effectively upon rehydration may be linked to their capacity to mitigate nitrosative stress. The management of water status during rehydration and the regulation of the nitrogen–nitrosative axis appear to be co-dependent factors that may contribute to wheat’s survival and recovery potential during early developmental stages.

The observed fluctuations in nitrite content provide critical insights into the differential drought tolerance mechanisms in tolerant and sensitive wheat seedlings. In both experimental groups, drought triggered a significant accumulation of NO_2_^−^, a phenomenon typically associated with the activation of the nitrate reductase pathway and the subsequent generation of nitric oxide (NO) [25]. However, the magnitude and duration of this accumulation varied substantially between the two developmental stages. The sensitive seedlings exhibited a more pronounced increase in nitrite levels than the tolerant ones. This suggests that the sensitive phenotype may exhibit a stronger accumulation of RNS precursors under water deficit, leading to prolonged nitrosative stress and protein nitration [27]. Significant differences were also evident during the recovery phase. Tolerant seedlings reduced nitrite levels to near-control values after rehydration, whereas sensitive seedlings maintained significantly elevated NO_2_^−^ concentrations, indicating a persistent nitrosative imbalance.

The inclusion of nitrate dynamics further elucidates the metabolic shift in wheat seedlings during the drought and recovery phases. The data indicate that both tolerant and sensitive seedlings accumulated NO_3_^−^ under a water deficit, though the relative increase was higher in sensitive plants than in tolerant plants. This increase in nitrate levels during drought is a typical response to decreased transpiration rates and the inhibition of nitrate reductase activity [25]. In sensitive seedlings, the sharp increase in nitrate content, coupled with the previously noted increase in nitrite levels, suggests a significant limitation in nitrogen assimilation. This imbalance may lead to an accumulation of substrate that the plant cannot efficiently process, potentially diverting nitrogen toward RNS production rather than amino acid synthesis [25]. In the recovery phase, a significant decrease in nitrate ions is observed in both groups, yet the sensitive seedlings exhibit a higher residual level than their initial state. This suggests that, while some assimilation resumes in 6-day-old plants, the re-establishment of the primary nitrogen reduction pathway is not fully effective. In tolerant seedlings, the lack of significant differences between treatments in nitrate levels suggests a more stable nitrogen metabolic flux. It may suggest the presence of mechanisms that prevent the excessive accumulation of nitrogenous precursors, which could otherwise contribute to nitro-oxidative stress [28]. When integrated with the nitrite and protein nitration data, these nitrate trends support the hypothesis that in 4-day-old wheat seedlings, nitrogen metabolism is less influenced by drought. Consequently, maintaining nitrate flux even under osmotic stress may facilitate drought tolerance.

Significant elevation of nitrates in response to drought in sensitive seedlings, with higher nitrite accumulation in sensitive plants after rehydration compared with tolerant seedlings, may suggest the occurrence of nitrosative stress. Nitrosative stress is likely to promote protein tyrosine nitration, particularly the formation of 3-nitrotyrosine, through NO-derived reactive nitrogen species such as peroxynitrite. Protein nitration may inhibit key metabolic enzymes and disrupt the photosynthetic apparatus [10]. In sensitive wheat seedlings, upregulated nitration of numerous proteins during recovery suggests that, as nitration usually reduces protein activity, it may contribute to their lack of drought resistance. The strong increase in protein nitration observed during the recovery phase in sensitive seedlings may be related to oxidative and nitrosative burst following rehydration [29]. The rapid restoration of metabolic activity can enhance the production of reactive oxygen and nitrogen species, including peroxynitrite, a key mediator of protein tyrosine nitration. In drought-sensitive seedlings, this effect may be intensified by reduced antioxidant and nitrosative stress-scavenging capacity [21]. Therefore, increased nitration during recovery in sensitive seedlings may reflect an imbalance in redox regulation and could contribute to impaired restoration of cellular functions after stress.

Conversely, the return of nitrites to control levels in the recovery group observed in 4-day-old seedlings and dominant downregulation of nitrated DAPs suggests that RNS homeostasis is not disturbed; therefore, if elevated, RNS may act primarily as signaling molecules at this developmental stage, promoting activation of antioxidant defenses without causing prolonged metabolic disruption associated with chronic protein nitration [30].

The proteomic analysis of tyrosine-nitrated proteins in tolerant wheat seedlings revealed that, although nitration is also considered a nitrostaive stress marker, several nitrated proteins are repeatedly downregulated. It may indicate that observed changes are not random byproducts of elevated RNS levels. In 4-day-old seedlings, the majority of the identified nitrated proteins (25 of 29) exhibited a significant decrease in their nitration levels during drought (ratio d/c < 1.0). This downward trend in nitration for key metabolic enzymes, such as ATP synthase, Rubisco, and carbonic anhydrase, suggests that the tolerant phenotype either actively denitrates these proteins, although the mechanism of denitration in plants is not proven [10,31], or degrades nitrated isoforms to maintain carbon assimilation and energy production under stress [10]. By reducing the abundance of nitrated proteins in the photosynthetic apparatus—evidenced by downregulation of the nitrated oxygen-evolving enhancer protein and ferredoxin–NADP reductase—the 4-day-old seedlings likely prevent the functional inhibition typically associated with 3-nitrotyrosine formation [32].

The specific dynamics of nitration in antioxidants and chaperone proteins further contribute to this adaptive strategy. For instance, 2-Cys peroxiredoxin and HSP70 show significantly reduced nitration during drought (d/c ratios of ~0.46–0.51), which may preserve their chaperone and peroxidase activities, ensuring that the plant’s defense systems remain fully functional even under elevated ROS and RNS levels [33,34]. On the other hand, the upregulated abundance of nitrated S-adenosylmethionine synthetase (SAMS) and mitochondrial ATP synthase subunit alpha (atp1-1) was observed during drought. The nitration of SAMS is particularly interesting. SAMS is a key enzyme in the methyl cycle and a precursor for ethylene and polyamine biosynthesis [35]. Its nitration could act as a metabolic switch, diverting resources toward specific stress-response pathways. Interestingly, in the recovery group, a few select proteins, such as SAMS and 14-3-3 proteins, show intense nitration (r/c ratios > 2.0). This increase suggests that nitration in tolerant seedlings may contribute to the inactivation of specific metabolic processes by inhibiting protein methylation, ethylene synthesis, and signal transduction [35,36]. In the recovery phase, it may contribute to post-stress developmental adaptations. In the STRING network analysis of 4-day-old tolerant wheat seedlings, the overwhelming predominance of downregulated nodes is visible during drought. This confirms that, in tolerant seedlings, there is no accumulation of 3-nitrotyrosine at critical metabolic hubs. The core of the network consists of densely connected proteins involved in energy production and carbon fixation, including ATP synthase subunits (atpA and B), the Rubisco large subunit (rbcL), phosphoglycerate kinase (PGKH), and ferredoxin–NADP reductase (A0A3B6NH85) [37,38,39]. Downregulation of these central nodes suggests that by reducing the nitration of these enzymes, the 4-day-old seedlings may maintain the functional integrity of their primary metabolism under a water deficit. The presence of nodes representing proteins whose nitration levels remain downregulated during recovery in the tolerant phenotype shows its ability to maintain function during rehydration. Notably, Heat Shock Protein 70 (TaHSP70d) and 2-Cys peroxiredoxin BAS1 (TSA) are downregulated nodes, suggesting that the plant’s chaperone and antioxidative systems are protected from nitration-mediated inhibition. HSP70 seems quite resistant to nitration under stress, and 2-Cys peroxiredoxin plays a protective role in chloroplasts against RNS [40,41]. It allows tolerant seedlings to remain fully operational for cellular repair and adaptation.

In stark contrast to the tolerant seedlings, the nitro-proteome profile of the 6-day-old (sensitive) plants reveals a greater proportion of nitrated proteins, particularly during the recovery phase. While some proteins show a decrease in nitration during the initial drought period—likely due to a general suppression of metabolic activity—the most notable phenomenon in sensitive seedlings is the upregulation of protein nitration upon rehydration (14 of 23 proteins). This recovery-induced nitration is particularly evident in essential energy-metabolism enzymes, such as various isoforms of ATP synthase and Rubisco activase, where r/c ratios often exceed 2.0. This suggests that sensitive 6-day-old seedlings may experience massive, secondary nitrosative stress during recovery. Protein nitration of key energy metabolism enzymes may directly affect the efficiency of metabolic recovery after drought stress. Enzymes such as ATP synthase and Rubisco activase are essential for ATP production and the maintenance of photosynthetic carbon assimilation; therefore, their nitration may potentially reduce enzyme activity and limit energy availability during the early phases of recovery [42]. The high nitration levels of Calvin cycle enzymes, including fructose-1,6-bisphosphate aldolase and sedoheptulose-1,7-bisphosphatase, during recovery indicate a severe impairment of CO fixation [43]. Impairment of carbon flux through the Calvin cycle may restrict the regeneration of photosynthetic capacity. Such modifications could delay the re-establishment of photosynthetic efficiency and overall metabolic homeostasis following rehydration [44]. Collectively, these changes may contribute to reduced recovery efficiency in sensitive seedlings by limiting both ATP supply and carbon assimilation, although further functional studies are required to confirm the direct impact of nitration on enzyme activity.

Unlike tolerant seedlings, which maintain low levels of protein nitration, sensitive seedlings exhibit numerous crucial enzymes modified by RNS. The nitration of actin (r/c ratio = 1.68) and aldehyde dehydrogenase (r/c ratio = 3.50) indicates a disruption of cellular structural integrity and detoxifying pathways [45,46]. This excessive accumulation of 3-nitrotyrosine during the transition from drought to recovery likely leads to either a loss of function or targeted degradation of the modified proteins, preventing the plant from resuming normal growth despite improved water availability [47]. Furthermore, the integration of these proteomic results with the physiological data suggests that in the recovery group of 6-day-old seedlings, elevated WSD in comparison to the control and higher nitrite content in comparison to tolerant seedlings may lead to the formation of chemical precursors necessary for peroxynitrite synthesis, which is the primary nitrating agent [48]. The massive post-drought nitration of the photosynthetic and mitochondrial apparatus may be a cause of drought susceptibility.

The STRING network analysis of sensitive wheat seedlings revealed a fundamentally different, more diverse nitrosative profile compared to that of tolerant seedlings, characterized by a high number of nitrated proteins during the transition from stress to recovery. The core of the interaction network comprises nitrated proteins upregulated in both drought and recovery states. Key central hubs include chloroplastic ATP synthase alpha subunit (atpA), sedoheptulose-1,7-bisphosphatase (D8L9G3), and vacuolar proton-ATPase subunit A (A0A3B6IPQ4). The simultaneous nitration of these hubs during drought and recovery suggests dysfunction of primary metabolism. Inhibition of atpA restricts energy supply, while modification of SBPase prevents its efficient use for carbon fixation [37,49]. Furthermore, V-ATPase impairment destabilizes vacuolar homeostasis, preventing the restoration of cell turgor [50]. These multi-organelle changes may be the cause of sensitive seedlings’ inability to recover upon rehydration, as the pathways for energy, carbon, and water balance are concurrently inhibited. Furthermore, the oxygen-evolving enhancer protein 2 (PSBP), Rubisco large subunit (rbcL), fructose-bisphosphate aldolase 8 (FBA8), mitochondrial ATP synthase subunit alpha (atp1-1), mitochondrial ATP synthase subunit alpha (atp1-1), and chloroplastic ATP synthase CF1 beta subunit (atpB) are primary targets of a nitrosative burst during rehydration, indicating that mitochondrial/chloroplastic energy apparatus is further inhibited [51,52]. It seems that the sensitive seedlings are unable to restrict nitration to a signaling role. Instead, it becomes a broad-spectrum inhibitory event.

## 4. Materials and Methods

The study was conducted on four- and six-day-old spring wheat seedlings (*Triticum aestivum* L. cv. Zadra) obtained from Plant Breeding Strzelce, a group of the Plant Breeding and Acclimatization Institute in Poland. Previous studies have shown that 4-day-old spring wheat seedlings are drought-resistant, while 6-day-old seedlings are sensitive to drought [4,21,22].

Surface-sterilized kernels were placed on paper rolls on Knop medium with Hoagland microelements and kept in the dark at 4 °C for 24 h for imbibition. The germinated kernels were then transferred to optimally controlled conditions in a growth chamber (16 h of light exposure at 260 mmol m^−2^ s^−1^ PPFD at 23 °C and 8 h in the dark at 16 °C, with a constant relative humidity of 70–80%). After 4 or 6 days, respectively, some seedlings were harvested and used as developmental-stage controls (control). These control samples represented the physiological baseline for each developmental stage prior to stress exposure. The remaining plants were subjected to drought stress via the removal of the liquid medium. The plants were brought to a WSD of approximately 50%, and another portion of the seedlings was harvested (drought). The remaining plants were rehydrated for 3 days and harvested (rehydration). The study was designed to compare stress-induced changes relative to the initial physiological state of drought-tolerant (4-day-old) and drought-sensitive (6-day-old) seedlings.

Physiological and biochemical analyses were performed with three independent biological replicates, whereas proteomic analyses were performed with four independent biological replicates. Each biological replicate consisted of three paper rolls, each containing 20 seedlings (60 seedlings per biological replicate). Material from multiple seedlings grown under identical conditions was pooled. Immediately after harvesting, all plants were frozen in liquid nitrogen and stored at −80 °C.

### 4.1. Water Saturation Deficit Measurement

The water content in the seedling shoots was measured as WSD according to Turner [53]. It was calculated using the following formula:

WSD (%) = (full turgor mass − actual fresh mass)/(full turgor mass − dry mass) × 100%

In this formula, the full turgor mass refers to the mass of the seedlings after overnight submersion in water in the dark, while the dry mass was determined after drying in an oven at 80 °C overnight. Three independent series of experiments were conducted. To measure shoot water content, each repetition included five randomly selected shoots.

In the proteomic experiment, well-watered shoots of wheat seedlings aged four and six days served as the control group. Seedlings of the same age were dehydrated to about 50% WSD to create a stressed group. Additionally, seedlings rehydrated for three days were included as the recovery group.

### 4.2. NO_3_^−^, and NO_2_^−^ Contents

NO_3_^−^, and NO_2_^−^ contents were determined using a modified method based on Huang et al. [54]. Leaf samples (100 mg FW) were incubated in 1 mL of deionized water with shaking at 45 °C for 1 h. Extracts were centrifuged at 16,000× *g* for 20 min at 4 °C, and the supernatants were collected for analysis. NO_3_^−^ and NO_2_^−^ concentrations were determined using the nitric oxide assay kit (Invitrogen/Thermo Fisher Scientific, Carlsbad, CA, USA) according to the manufacturer’s instructions. Absorbance was measured at 540 nm, and concentrations were calculated from standard curves and expressed as µM g^−1^ FW.

### 4.3. Statistical Analysis

All data were subjected to statistical analyses. Two-way ANOVA and Tukey’s test were performed using MS-Excel 2010 ( Microsoft Corporation, Redmond, WA, USA) and the Statistica 12 software (TIBCO Software Inc., Palo Alto, CA, USA). Applied *p*-values are given under the individual figures. All measurements were performed in 3 biological replicates (*n* = 3).

### 4.4. Preparation of Total Protein Extracts

To analyze the proteome profile of nitrated proteins, the systematic extraction and purification of proteins was conducted. Tissue samples from above-ground plant shoots were homogenized into a fine powder in liquid nitrogen. A 150 mg aliquot of pulverized tissue was transferred to a 2 mL test tube and purified according to the protocol by Wang et al. [55].

Protein precipitation was initiated by the addition of ice-cold 10% (*w*/*v*) trichloroacetic acid (TCA) in acetone. The sample was mixed thoroughly and incubated at −20 °C for 30 min. After incubation, the mixture was centrifuged at 16,000× *g* for 30 min at 4 °C, and the supernatant was carefully discarded. The resulting pellet underwent two washes with ice-cold TCA/acetone.

Next, the tube was filled with 80% methanol containing 0.1 M ammonium acetate, and the mixture was briefly incubated before centrifuging for 15 min (16,000× *g*, 4 °C). The supernatant was removed, and the pellet was washed with 80% acetone, mixed again, and centrifuged, with the supernatant discarded. The remaining pellet was air-dried at room temperature to eliminate residual acetone and subsequently dissolved in 0.6 mL of phenol (pH 8.0) and 0.6 mL of SDS buffer (0.1 M Tris-HCl, pH 8.0, containing 30% (*w*/*v*) sucrose, 5% (*v*/*v*) β-mercaptoethanol, and 2% (*w*/*v*) SDS). Following a 5 min incubation at room temperature, the mixture was centrifuged at 16,000× *g* for 15 min at 4 °C, and the upper (phenolic) phase was collected into a new tube.

Protein extraction was finalized by precipitating the proteins overnight at −20 °C in 1.5 mL of 0.1 M ammonium acetate in 80% methanol. Post-centrifugation for 30 min, the supernatant was discarded, and the protein pellet was thoroughly washed with pure methanol and 80% acetone. The air-dried pellet was then re-solubilized in an isoelectric focusing (IEF) buffer composed of 7 M urea, 2 M thiourea, 4% (*w*/*v*) CHAPS, and 40 mM dithiothreitol (DTT). The protein concentration was determined photometrically at 595 nm using the Bradford method [56].

### 4.5. Two-Dimensional Gel Electrophoresis, Protein Transfer, and Image Analysis

Purified proteins were separated using two-dimensional gel electrophoresis (2D-PAGE). Initially, the precipitated proteins (20 μg) were dissolved in 125 μL of an isoelectric focusing (IEF) buffer, which consisted of 7 M urea, 2 M thiourea, 4% (*w*/*v*) CHAPS, 40 mM DTT, and 0.5% (*v*/*v*) pH 3–10NL ampholytes, along with bromophenol blue. These protein mixtures were then subjected to IEF on 7 cm pH 4–7NL Immobiline DryStrips (Bio-Rad, Hercules, CA, USA), utilizing the Bio-Rad PROTEAN IEF chamber flatbed electrophoresis system, as specified by the manufacturer’s guidelines.

Following the IEF procedure, the strips were incubated in an equilibration buffer (50 mM Tris-HCl, pH 8.8, 6 M urea, 30% (*v*/*v*) glycerol, 2% (*w*/*v*) SDS, and a hint of bromophenol blue), first with 1% (*w*/*v*) DTT for 10 min, and subsequently in a buffer with 2.5% (*w*/*v*) iodoacetamide for an additional 10 min. Once equilibrated, the strips were meticulously placed atop the SDS-PAGE gels, comprising a 4% concentrating gel and an 11% separating gel (8.6 × 6.8 cm × 0.1 cm), using 0.5% (*w*/*v*) agarose in 0.1 M Tris-HCl, pH 6.8, and bromophenol blue. The SDS-PAGE was conducted in a 50 mM Tris-HCl buffer, pH 6.8, at a constant amperage of 20 mA per gel for approximately 1 h, or until the blue dye front reached the bottom of the gel.

### 4.6. Detection of Nitrated Proteins

To detect proteins containing 3-nitrotyrosine (3-NT) after two-dimensional electrophoresis, the proteins were transferred to a polyvinylidene fluoride (PVDF) membrane using a Bio-Rad transfer system according to the manufacturer’s protocol.

The membrane was blocked by incubating it for 1 h at room temperature with shaking in 5% (*w*/*v*) non-fat milk dissolved in a TBS buffer (pH 7.4) with 0.5% (*v*/*v*) Tween 20. After washing the membrane three times with a TBS buffer containing Tween 20 and once with a TBS buffer without Tween 20, the proteins were detected by incubating the membrane with a rabbit anti-3-nitrotyrosine primary antibody (Sigma-Aldrich, St. Louis, MO, USA; diluted 1:1000). This was followed by another wash (as previously described) and incubation with alkaline phosphatase-conjugated goat anti-rabbit antibodies (Sigma-Aldrich; diluted 1:30,000). The blots were visualized using a standard NBT/BCIP solution containing 0.1 M Tris-HCl (pH 9.5), 0.1 M NaCl, 0.05 M MgCl_2_, and 1.5 mg of BCIP and 3 mg of NBT. Four biological replicates were performed for each experimental variant, including protein purification, 2DE, membrane transfer, and immunochemical staining (*n* = 4).

Additionally, preparative 2D gels were used for each experimental variant. Proteins were purified and separated using 2DE as previously described, but they were not transferred to a PVDF membrane. The resulting gels were stained with colloidal Coomassie.

PVDF membranes were digitalized with the G: BOX EF2 system (Syngene, Bangalore, Karnataka, India), and their digital images were analyzed using the 2D gel analysis software Delta2D Version 2.0 (DECODON GmbH, Greifswald, Germany). Differential proteins were detected by comparing dehydrated and rehydrated seedlings with controls within seedlings with the same level of dehydration tolerance. Normalized spot intensities were yielded by relating the single spot intensity to the total spot intensity of all detected spots in the membrane image. The accuracy of membrane identity between biological replicates was evaluated using principal component analysis, which served both as a quality control step and as a means to compare all experimental variants. Heatmaps and hierarchical clustering analyses were generated using Delta2D Version 2.0 (DECODON GmbH, Greifswald, Germany) from normalized spot intensities obtained from the membrane images. Spot intensities were normalized to the total intensity of all detected spots in a given membrane. Hierarchical clustering was performed using Euclidean distance, and the resulting heatmaps were used to visualize similarities and differences in protein nitration profiles among experimental groups. The selection of differential proteins was based on mean spot intensity, and the results were evaluated using one-way ANOVA with an adjusted Bonferroni correction (*p*-value < 0.05). Images of selected spots on membranes were overlaid with the corresponding gel images, and the spots were then cut out from the gels for identification.

### 4.7. Protein Sample Preparation and Identification

Isolated protein spots were primarily identified by LC–MS/MS analysis. The spots were cut from the gel, incubated in 100 μL of destaining solution (50% (*v*/*v*) 50 mM ammonium bicarbonate and 50% (*v*/*v*) ACN), dried, and incubated in 100 μL of pure ACN until they shrank. Then, the Cys residues were reduced by incubating the gel pieces in 50 μL of 10 mM DTT dissolved in 100 mM ammonium bicarbonate. After drying and shrinking the gel pieces as described above, the Cys residues were alkylated by incubating the gel pieces in 50 μL of 50 mM iodoacetamide in 100 mM ammonium bicarbonate. The gel pieces were washed twice with 100 mM ammonium bicarbonate and dried in pure ACN. After shrinking the gel pieces in pure ACN, trypsin digestion was performed overnight at 37 °C (10 ng μL^−1^ in 25 mM ammonium bicarbonate). The peptides were extracted (three repetitions) from the gel pieces with a solution containing 0.1% TFA (*v*/*v*) and 2% (*w*/*v*) ACN.

The NCBIprot 20191214 database (5 November 2020, 229,636,095 sequences; 83,676,080,993 residues) *Triticum aestivum* taxonomy (19,342 sequences) was used, and MASCOT score results above the threshold *p* < 0.05 were considered probable. The peptide mass tolerance was set at ±20 ppm, with a fragment mass tolerance of ±0.1 Da and an allowed missed cleavage of up to 1.

### 4.8. Bioinformatics Protein Interaction Data Analysis

The prediction of functional networks of nitrated proteins was performed using the STRING database (http://string-db.org, accessed on 2 April 2026), which contains known and predicted protein interactions [57]. Network mapping was performed using default parameters, including a medium-confidence score threshold of 0.400 and integration across multiple evidence channels. The resulting interaction networks were exported into Cytoscape software (v3.10.4) for topological visualization and analysis. To improve clarity and emphasize functional modules, isolated nodes representing proteins without predicted interactions were removed from the final network visualizations.

## 5. Conclusions

In conclusion, drought sensitivity in 6-day-old wheat may be associated with an inability to restrain nitrosative stress during recovery, leading to extensive protein nitration, persistent metabolic disruption, and progressive cellular decline. When considered together with the data on water status and nitrogen metabolism, these results suggest that drought tolerance in 4-day-old wheat may depend on a more effective balance between protein nitration and removal of nitrated proteins. In contrast to the sensitive 6-day-old seedlings, which likely experience increased protein nitration in the recovery phase due to an elevated water saturation deficit after rehydration and greater nitrite accumulation compared with tolerant seedlings, the 4-day-old seedlings appear to maintain lower nitration of crucial proteins. The ability to limit nitration of primary metabolic and defense-related proteins during the drought may therefore represent an important physiological requirement for survival under a water deficit, enabling more rapid metabolic recovery upon rehydration. Overall, this mechanism may contribute to drought tolerance during early wheat development; however, further functional validation is required to confirm the direct impact of protein nitration on protein function and stress tolerance.

## Data Availability

The original contributions presented in this study are included in the article/Supplementary Material. Further inquiries can be directed to the corresponding author.

## Supplementary Materials

The following supporting information can be downloaded at https://www.mdpi.com/article/10.3390/plants15131951/s1, Figure S1: Representative Western blot membrane images showing nitrated proteins in 4-day-old (tolerant) wheat seedlings. Numbered spots indicate proteins selected for identification by mass spectrometry following image analysis in Delta2D software Version 2.0 (DECODON GmbH, Greifswald, Germany). Figure S2: Representative Western blot membrane images showing nitrated proteins in 6-day-old (sensitive) wheat seedlings. Numbered spots indicate proteins selected for identification by mass spectrometry following image analysis in Delta2D software Version 2.0 (DECODON GmbH, Greifswald, Germany). Table S1: Normalized spot quantities of nitrated proteins detected in 4-day-old drought-tolerant wheat seedlings under control, drought, and recovery conditions. Statistical significance was determined using one-way ANOVA followed by Bonferroni correction (*p* ≤ 0.05). Spot IDs correspond to the protein spots identified by mass spectrometry and shown in the corresponding Western blot analyses. Table S2: Normalized spot quantities of nitrated proteins detected in 6-day-old drought-sensitive wheat seedlings under control, drought, and recovery conditions. Statistical significance was determined using one-way ANOVA followed by Bonferroni correction (*p* ≤ 0.05). Spot IDs correspond to the protein spots identified by mass spectrometry and shown in the corresponding Western blot analyses.
