# Supplementary figures and images for: The Wheat Nitro-Proteome: Protein Nitration Profiles During Drought and Rehydration
Source: Plants (Basel). 2026 Jun 24;15(13):1951. doi: 10.3390/plants15131951 (PMC13364139; doi:10.3390/plants15131951)

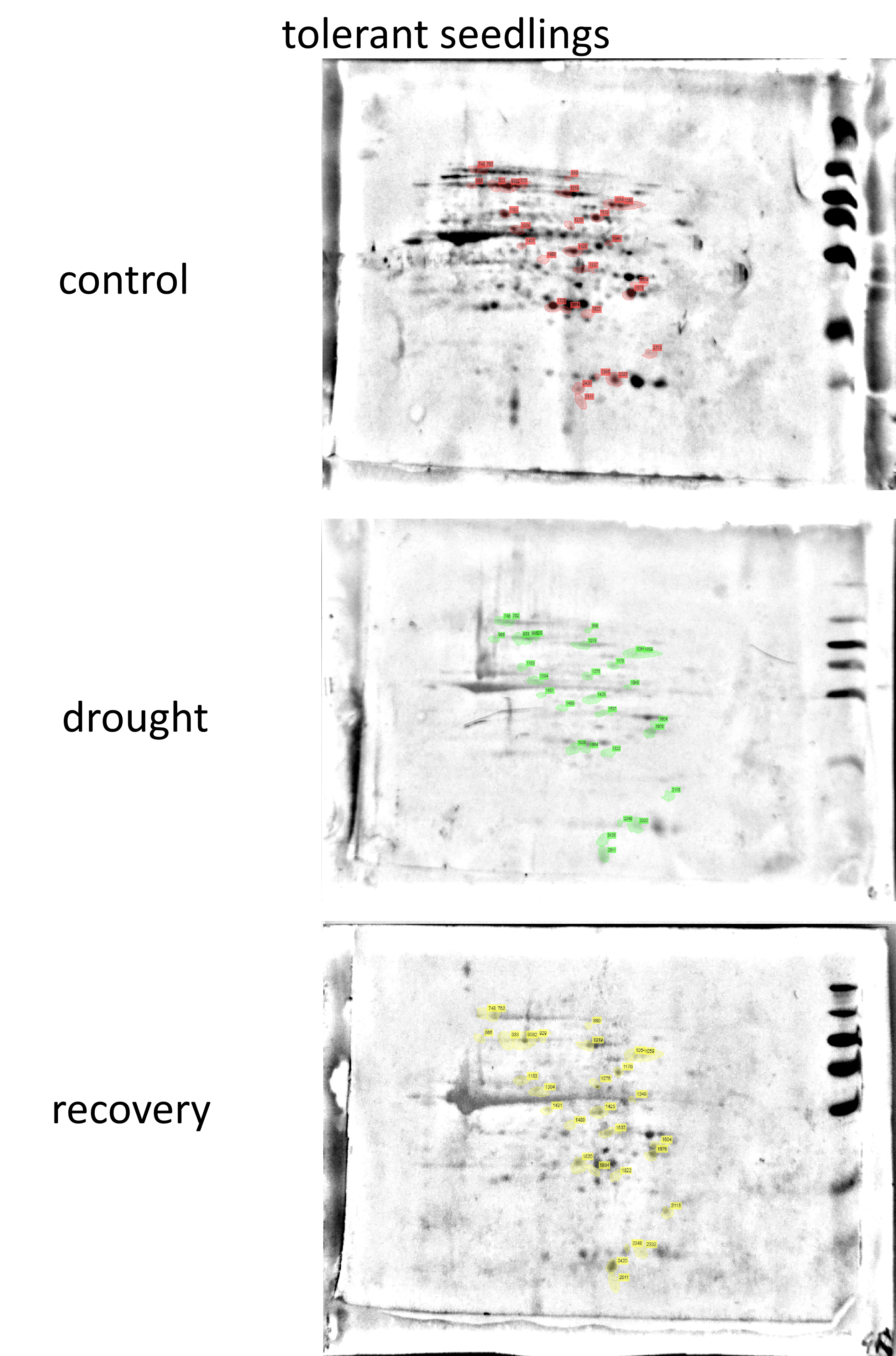

Supplement: Supplementary file 1 [file plants-15-01951-s001.zip › plants-4376662-supplementary/Figure S1.png]

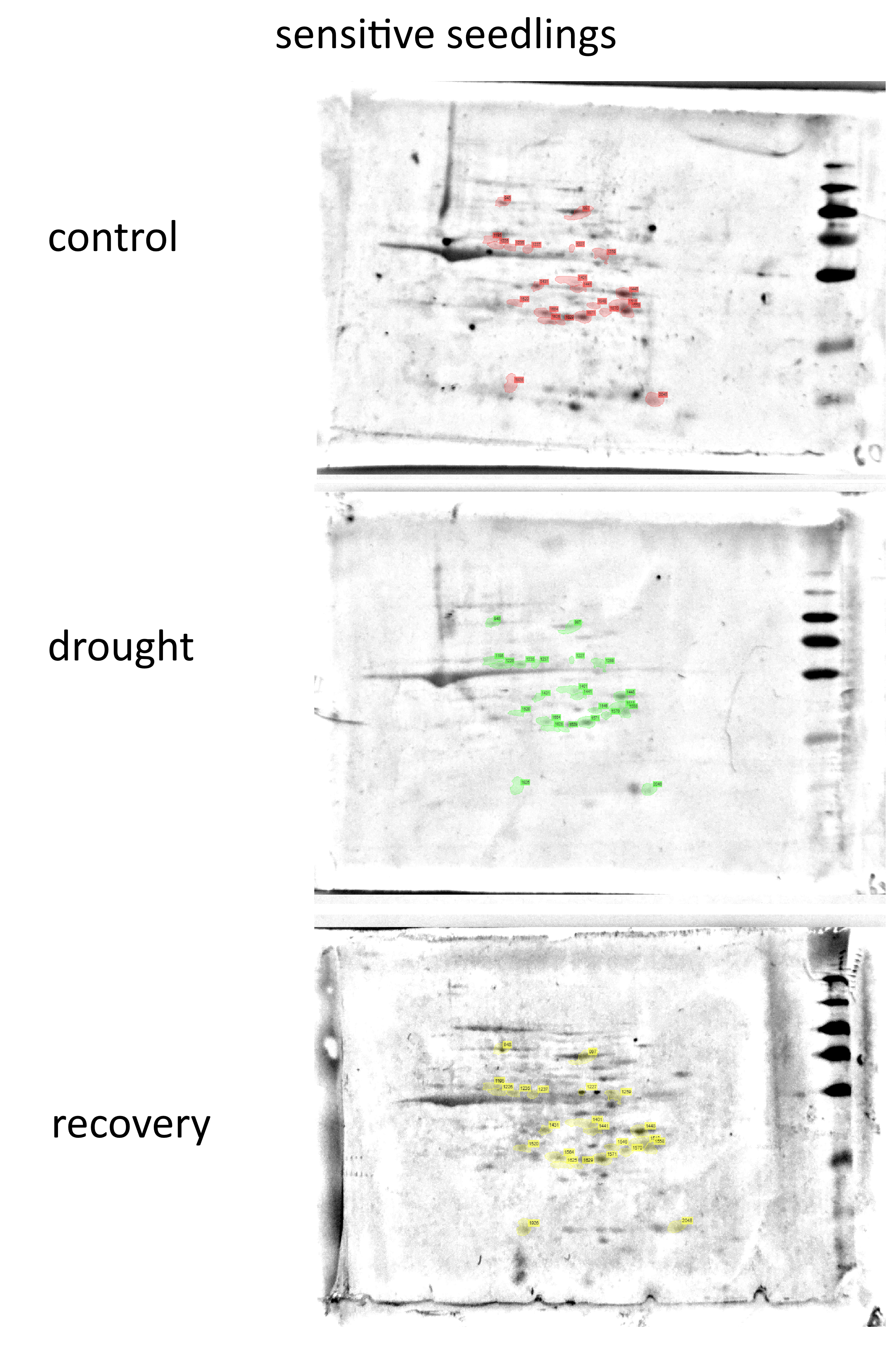

Supplement: Supplementary file 1 [file plants-15-01951-s001.zip › plants-4376662-supplementary/Figure S2.png]
